# Dynamic reconfiguration of cortical functional connectivity across brain states

**DOI:** 10.1038/s41598-017-08050-6

**Published:** 2017-08-18

**Authors:** Iain Stitt, Karl J. Hollensteiner, Edgar Galindo-Leon, Florian Pieper, Eva Fiedler, Thomas Stieglitz, Gerhard Engler, Guido Nolte, Andreas K. Engel

**Affiliations:** 1 Department of Neurophysiology and Pathophysiology, University Medical Center Hamburg-Eppendorf, 20246 Hamburg, Germany; 2 Department of Psychiatry, Neuroscience Center, University of North Carolina, Chapel Hill, NC 27514 USA; 3 Department of Microsystems Engineering, University of Freiburg, 79110 Freiburg, Germany

## Abstract

Throughout each day, the brain displays transient changes in state, as evidenced by shifts in behavior and vigilance. While the electrophysiological correlates of brain states have been studied for some time, it remains unclear how large-scale cortico-cortical functional connectivity systematically reconfigures across states. Here, we investigate state-dependent shifts in cortical functional connectivity by recording local field potentials (LFPs) during spontaneous behavioral transitions in the ferret using chronically implanted micro-electrocorticographic (µECoG) arrays positioned over occipital, parietal, and temporal cortical regions. To objectively classify brain state, we describe a data-driven approach that projects time-varying LFP spectral properties into brain state space. Distinct brain states displayed markedly different patterns of cross-frequency phase-amplitude coupling and inter-electrode phase synchronization across several LFP frequency bands. The largest across-state differences in functional connectivity were observed between periods of presumed slow-wave and rapid-eye-movement-sleep/active-state, which were characterized by the contrasting phenomena of cortical network fragmentation and global synchronization, respectively. Collectively, our data provide strong evidence that large-scale functional interactions in the brain dynamically reconfigure across behavioral states.

## Introduction

Across the sleep/wake cycle we typically transition between states of varying vigilance and unconsciousness. Such transitions in behavioral state (conscious vs asleep) are correlated with changes in spatiotemporal patterns of neural activity, reflecting the close link between behavioral state and brain state across the sleep/wake cycle. The electrophysiological correlates of various brain states have been extensively studied with recordings of the electroencephalogram (EEG), local field potentials (LFP), and cellular activity throughout the past 60 years. Accordingly, brain states have been increasingly defined based on the presence of distinct patterns of oscillatory neural activity^1^. Although these approaches have provided a wealth of knowledge on brain state dependent fluctuations in local neuronal activity, recent studies have shifted towards identifying the spectral signatures of large-scale functional interaction in the brain^2, 3^. One of the motivations for this shift toward investigating the functional connectivity of different brain states has been empirical and theoretical work suggesting that the complexity of interactions between functionally specialized brain regions is a key mechanism related to consciousness^4–10^. However, it remains unclear how such interactions between functionally specialized brain regions dynamically reconfigure across brain states.

The majority of previous studies on large-scale functional connectivity are confounded by the fact that subjects tend to spontaneously drift between distinct brain states throughout the duration of recordings^11–13^, leading some to call for a renewed approach that captures the true time-varying nature of inter-areal communication by discarding the assumption that functional connectivity is spatiotemporally invariant^14, 15^. Under normal circumstances, the most drastic transitions in state, both in terms of behavior and neural activity, occur throughout alternations between wakefulness and sleep. Our aim in this study was to investigate cortical functional connectivity in a time-resolved manner as ferrets spontaneously transition between brain states. Given that the dynamic formation and fragmentation of large-scale brain networks is proposed to underlie consciousness, we hypothesize that transitions in brain state lead to dynamic reorganization of both local and long-range cortico-cortical functional connectivity. To test this hypothesis, we recorded LFPs during spontaneous behavior, including movement, quiet awake, and sleep periods, using custom micro-electrocorticographic (µECoG) arrays chronically implanted in ferrets. The advantage of this approach was that, in contrast to humans, ferrets alternate relatively quickly across sleep, resting, and awake states^16, 17^. This enables us to sample neural data from many consecutive sleep/awake cycles with relatively short recordings. In contrast to previous approaches that subjectively classify brain state into several discrete states based on electrophysiological and electromyographic signals, we employ an approach that captures the most relevant time-varying components of µECoG signals, and project these as continuous variables into state space. Brain states in this study were thus exclusively classified based on electrophysiological signatures of neural activity detected in cortex. Crucially, this state-classification method enabled the examination of how patterns of large-scale cortical functional connectivity evolve as animals traverse through brain state space. Our data reveal that patterns of cortical synchronization systematically reconfigure across brain states, with the most striking across-state changes occurring between periods of presumed slow-wave and rapid eye movement sleep.

## Results

**Figure 1:**
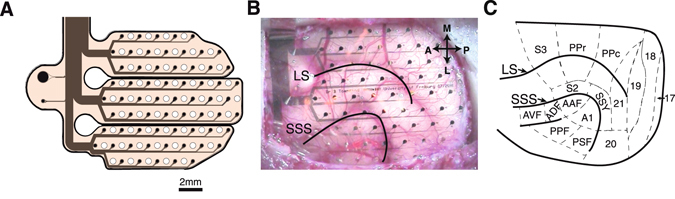
µECoG design and placement over ferret posterior cortex. (**A**) The µECoG consists of 64 platinum electrodes of 250 µm diameter distributed across three polymide fingers to enable the array to adapt to the curved surface of the cortex. Inter-electrode spacing was 1.5mm. (**B**) Placement of custom designed µECoG array over the left hemisphere during implantation of animal #3. (**C**) Ferret posterior cortex parcellated into 16 functionally and anatomically distinct regions according to Bizley *et al*. (2007)^53^). Abbreviations: LS: lateral sulcus; SSS: suprasylvian sulcus; A: anterior; P: posterior; M: medial; L: lateral; S2/S3: somatosensory cortex 2 & 3; PPr/c: rostral/caudal posterior parietal cortex; 17,18,19,20,21: occipital/visual cortex; SSY: suprasylvian field; A1: primary auditory cortex; AAF: anterior auditory field; APF: anterior dorsal field; AVF: anterior ventral field; PPF: posterior pseudosylvian field; PSF: posterior suprasylvian field.

We recorded cortical surface LFPs from 5 ferrets that were chronically implanted with 64 channel custom designed µECoG arrays (Fig. 1). The spacing of the µECoG array electrodes (1.5 mm) ensured that we sampled data from vast areas of occipital, parietal, and temporal cortex simultaneously. To assess large-scale cortical dynamics associated with spontaneous brain state transitions, animals were recorded across multiple sessions with a minimum length of 2 hours each. The short sleep/wake cycle of the ferret^16, 17^ ensured that we captured several brain state transitions during each recording session.

### Projection of time-varying spectral properties into state space

**Figure 2:**
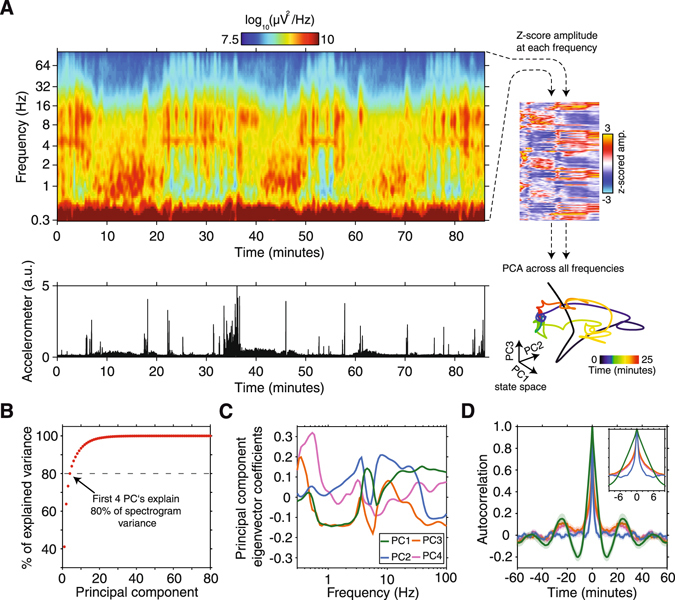
State-dependent fluctuations in spectral power and projection of neural data into PCA-derived state-space. (**A**) A representative example µECoG power spectrogram from an electrode placed over visual cortical area 21. Accelerometer power is plotted below the spectrogram to show how animals behavioral state changed over the duration of the recording. Note the recurring fluctuations in LFP power in the delta (0.8–3 Hz), theta (4–6 Hz), alpha (8–16 Hz), and gamma (30–80 Hz) frequency bands that track transitions in animal state. PCA was performed on the z-scored amplitude spectrogram (upper right, 90° rotated) to capture state-dependent fluctuations in LFP amplitude and project data into state-space (lower right). (**B**) The amount of variance explained by each principal component for the representative recording used to compute PCA coefficients. The first four principal components cumulatively explain more than 80% of total spectrogram variance (**C**) Principal component eigenvector coefficients for the first four principal components as a function of LFP frequency. Note that each component captures state-dependent fluctuations in LFP amplitude across several frequency bands. (**D**) The mean autocorrelation of principal components across all recording sessions and animals (± SEM). The prominent side peaks of principal components 1, 2 & 3 at ± 23 minutes reflect the periodic nature of spectral properties captured by these components, and match the reported duration of sleep/wake cycles. In contrast, principal component 2 displayed no side peaks and a much shorter half-width (blue trace), indicating that this component captures rapid and aperiodic fluctuations in spectral properties.

To assess how LFP spectral characteristics evolve across spontanius behavioral recordings, we computed sliding-window estimates of µECoG signal power across each recording session (Fig. 2A). Cortical LFP power spectra displayed systematic and recurring fluctuations that tracked spontaneous shifts in behavioral state, as measured by animal movement. For example, while animals were alert and moving about, LFP spectra were typically characterized by theta (4–6 Hz) oscillations coupled with relatively low delta oscillation power (0.8–3 Hz). In contrast, when animals were stationary µECoG spectra tended to display strong delta and low gamma (30–80 Hz) power (Fig. 2A). To capture the spectral signatures that most reliably reflect transitions in brain state throughout recordings, we computed principal component analysis (PCA) on the z-score normalized LFP amplitude spectrogram (Fig. 2A, right). This step reduces dimensionality of spectrograms by capturing state-dependent covariance across all LFP frequencies. The new principal component coordinate system therefore maximally captures state-dependent fluctuations in LFP signal amplitude. The first four principal components collectively explained more than 80% of spectrogram variance (Fig. 2B). Therefore, only the first four principal components were used for construction of state-space and subsequent analysis steps.

By examining principal component eigenvector coefficients as a function of LFP frequency, one can gain an intuitive understanding of how each principal component is derived from the input spectrogram (Fig. 2C). For example, the first principal component predominantly captures fluctuations in the amplitude of delta, theta, and gamma bands, whereas the second principal component captures fluctuations in the amplitude of alpha and gamma rhythms. Principal components are derived by finding eigenvectors that maximize variance across multidimensional datasets. Therefore, performing PCA on each individual recording session would lead to varying estimates of PCA eigenvector coefficients. To ensure that spectral amplitudes from all animals were translated into an equivalent state space coordinate system, we performed PCA on data from one animal as a training dataset. The resulting PCA eigenvector coefficients were then convolved with z-scored spectral amplitude matrices from all other sessions and animals. To illustrate that principal components systematically fluctuate in a timeframe consistent with the duration of the ferret sleep/wake cycle, we computed the autocorrelation of the first four principal components across all recording sessions. The first, third, and fourth principal components displayed prominent autocorrelation side peaks at approximately 23 minutes (Fig. 2D), indicating that spectral signatures captured by these components display periodicity in a time scale that is consistent with the average duration of the ferret sleep/wake cycle^16, 17^. In contrast to other components, the second principal component displayed no side peaks and a sharper autocorrelation peak (returning to ~0 at ± 2 minutes), indicating that spectral signatures captured by this component fluctuate on a much shorter timescale (Fig. 2D, blue trace). Now that we have shown that principal component analysis can be used to project time-varying spectral properties into brain state space, the next step was to objectively cluster data represented in state space into an optimal number of brain states.

### Clustering of brain states in PCA-derived state-space

**Figure 3:**
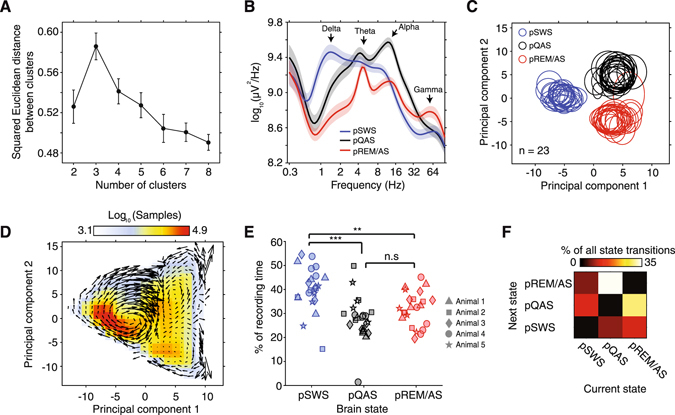
Objective classification of brain state by k-means clustering of data in state-space. (**A**) Data were clustered using a k-means clustering algorithm with the initial assumption of between 2 to 8 data clusters. Squared Euclidean distance between resultant clusters was maximal assuming 3 clusters, indicating that state/space data is optimally classified into 3 brain state clusters (error bars ± SEM). (**B**) The population average power spectrum computed across clustered brain states. Based on spectral characteristics of each state, clustered brain states were putatively labeled either presumed SWS (blue trace, slow wave sleep), quiet awake (black trace, pQAS), or rapid-eye-movement/active-state (red trace, pREM/AS). (**C**) The distribution of brain state data clusters across the first two principal components for all recording sessions and animals (n = 23). The mean of each cluster in state space is represented by the center of each ellipse, while standard deviation is represented by eccentricity along each axis. Note that brain states consistently localize in the same regions of state space across recordings and animals. (**D**) The number of samples binned across the first two components of state space provides a heat map of where animals spent the most time in state/space. Velocity vectors were overlaid to show the mean trajectories animals traversed through state space. (**E**) The percentage of time spent in each clustered sleep/wake state for each recording session. **p < 0.01, ***p < 0.001, kruskal-wallis test, Bonferroni corrected p-values. (**F**) The population average rate of transitions between brain states. Note that the most frequency state transitions occurred between pREM/AS and pQAS, which are maximally separated fluctuations along the second principal component axis.

We computed the optimal number of clusters across brain state space by clustering data with a k-means clustering algorithm assuming between 2 to 8 clusters (Fig. 3A). The optimal number of state-space clusters was determined by computing the squared Euclidean distance between all resultant data clusters for each assumed number of clusters. Optimal separation of state-space clusters occurred with the initial assumption of 3 data clusters (Fig. 3A). Therefore, we clustered all state-space data into 3 clusters. To relate each state space cluster to sleep/wake states that have been scored using polisomnography, we compared LFP power spectra during each state to data from Jha *et al*. (2006)^17^. Based on this comparison, we classified resultant states as presumed slow-wave-sleep (pSWS), presumed quiet-awake state (pQAS), and presumed rapid-eye-movement-sleep/active-state (pREM/AS). By plotting the distribution of clustered data across the first two components of PCA-derived state-space, one can observe that classification of brain states is consistent across different recording sessions and animals (Fig. 3C).

To show how animals traversed state space over time, we computed the first derivative across each of the first two principal components, and combined this information to construct velocity vectors in state-space (Fig. 3D). This approach revealed characteristic trajectories between all brain states. For example, when observed across the first two principal components, animals first entered the pSWS cluster from the lower right quadrant, before circulating in a clockwise fashion through the pSWS cluster to finally exit from the upper right quadrant (Fig. 3D). Similar characteristic trajectories were observable for the other brain states. Collectively, state-space trajectories support the hypothesis that resting/awake brain states are best represented by a continuum of constantly fluctuating neuronal variables, with states overlapping and flowing into one another^18^.

To uncover the regions of state-space where animals spent the most time, we divided the first two principal components of state space up into bins and counted the number of data samples that fell into each bin (Fig. 3D). This analysis revealed that animals tended to dwell for the most time in the lower half of the pSWS cluster. This was confirmed by computing the percentage of time spent in each brain state data cluster, with animals spending 40.3 ± 1.9% of time in the pSWS state, 31.9 ± 1.5% in the pREM/AS, and 27.8 ± 1.9% in the pQAS (all errors are ±standard error mean). In line with this, animals spent significantly more time in the pSWS state than other states (Fig. 3D, p < 0.01 Kruskal-Wallis test, Bonferroni corrected). Despite spending the most time in the pSWS state, the most common state transitions occurred between the pREM/AS and pQAS (pREM/AS → pQAS 29.5 ± 6.1%, pQAS → pREM/AS 34.4 ± 7.2% of all transitions, respectively, Fig. 3F, all sessions Supplementary Figure 2). Transitions between pQAS and pREM/AS states were more frequent because these states are predominantly separated by variance in spectral amplitude captured by the second principal component, which was earlier shown to fluctuate on a shorter time scale to other components (Fig. 2D).

**Figure 4:**
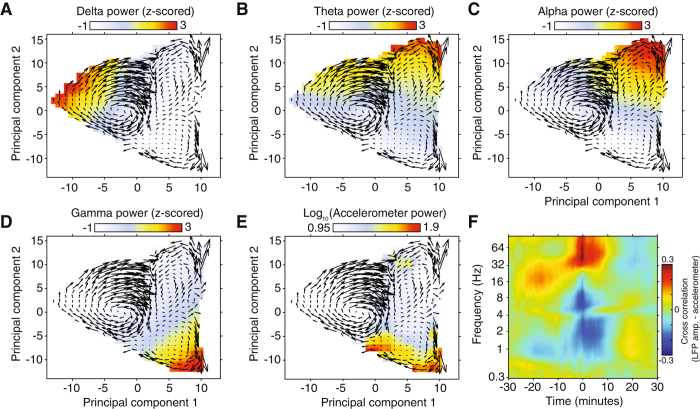
Distribution of LFP and accelerometer power across state space. (**A**–**D**) Plots represent the session averaged LFP power in delta (**A**), theta (**B**), alpha (**C**), and gamma (**D**) frequency bands across the first two principal components that constitute sleep/wake state space. Overlaid on each plot are mean state space trajectories, illustrating how the brain continuously traverses between sleep/wake states. (**E**) Displays the same as plots **A**–**D**, but with accelerometer power. (**F**) The population average cross-correlation of LFP amplitude and accelerometer power, resolved by LFP frequency. Note that animal movement coincides with periods of elevated high frequency amplitude (> 30 Hz), and reduced low frequency amplitude (< 14 Hz).

Figure 4 displays how spectral power in the delta, theta, alpha, and gamma frequency bands fluctuates across state space. Given that each principal component captures covariance across several frequency bands, these plots help to form an intuitive understanding of how the amplitude of signals in these physiologically relevant frequency bands are modulated across state space. For comparison with animal behavior, we have also plotted the mean accelerometer across state space (Fig. 4E), and the cross-correlation of µECoG signal amplitude and accelerometer power (Fig. 4F). Periods of movement corresponded to moments of elevated gamma power and reduced low-frequency (<16 Hz) power. One interesting aspect of these results is that our data driven brain state classification approach seems to have clustered epochs of REM and active awake in the same state, reflecting that spectral signatures of these states are inherently similar.

### Cross-frequency interactions across brain states

Cross-frequency phase amplitude coupling (PAC) has been proposed as a key mechanism for synchronizing neural networks across multiple spatial and temporal scales^19^. Given that fluctuations in the spectral signatures of neural activity can be used to define brain state, we next asked if each clustered brain state displayed different signatures of cross-frequency interaction. To assess cross-frequency coupling we computed bicoherence, a third order spectral measure that quantifies the relationship between three frequencies. In practice, results for bicoherence are very similar to those of PAC. While for bicoherence the dependence on three frequencies is explicit, with the third frequency constrained to be the sum of the first two, this dependence is implicit in PAC and is contained in the requirement that the filter for the high frequency band must be relatively wide and is recommended to be at least twice as large as the lower frequency^20^. Such filter settings for PAC, which require re-filtering the data with a different filter for each low frequency, are not only a large computational burden, but such filters also cause smearing across frequencies. In particular, in contrast to PAC, for bicoherence higher harmonics of narrow band rhythms occur as sharp peaks and can be distinguished easily from interactions between wider bands.

**Figure 5:**
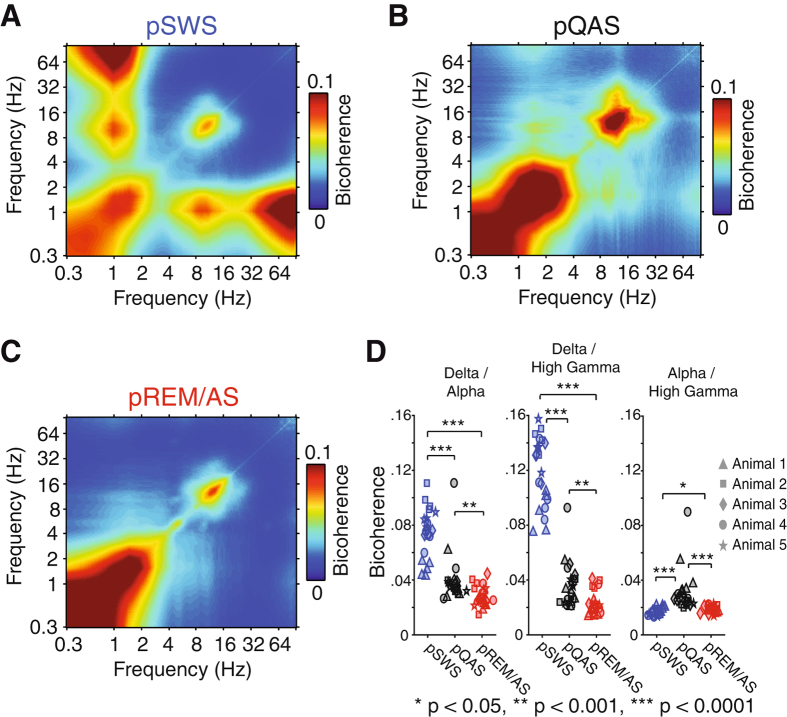
State dependent changes in phase amplitude coupling measured using bicoherence. (**A**) Population average bicoherence spectrum during the pSWS state. Note the prominent coupling between delta (~1 Hz) and alpha (8–16 Hz), as well as delta and high gamma (60–200 Hz) frequencies. In addition, delta and alpha oscillations are coupled to their first harmonic. (**B**) Population average bicoherence spectrum during the pQAS state. Note the weak coupling between alpha (8–16 Hz) and high gamma (60–200 Hz) frequencies. (**C**) Population average bicoherence spectrum during the pREM/AS state. In contrast to other states, pREM/AS displays fewer cross frequency interactions. (**D**) The strength of bicoherence for identified frequency-frequency interactions for each recording session (*p < 0.05, **p < 0.001, ***p < 0.0001, Kruskal-Wallis test, Bonferroni corrected p-values).

State-dependent bicoherence analysis revealed several cross-frequency PAC bands that varied as a function of resting/awake brain state (Fig. 5). Strongest bicoherence was measured between delta oscillations (~1 Hz) and high gamma (60–200 Hz) during the pSWS state (bicoherence 0.120 ± 0.025 SD). The strength of delta/high gamma bicoherence was significantly reduced in the pREM/AS and pQAS (pREM/AS bicoherence = 0.023 ± 0.009 SD, pQAS bicoherence = 0.036 ± 0.016 SD, p < 0.0001 Kruskal-Wallis test, Bonferroni corrected). We similarly observed strong coupling between delta and alpha oscillations in the pSWS state (Fig. 5A) that was significantly reduced in other brain states (pSWS bicoherence = 0.075 ± 0.018 SD, pQAS bicoherence = 0.040 ± 0.017 SD, pREM/AS bicoherence = 0.028 ± 0.007 SD, p < 0.0001 Kruskal-Wallis test, Bonferroni corrected). Cross-frequency coupling for the delta/high gamma and delta/alpha frequency bands likely reflects the phase locking of spiking activity and spindles to the up state of cortical slow oscillations during the pSWS state. In addition to these frequency bands, we observed weak alpha/high gamma bicoherence that was strongest in the pQAS state (Fig. 5B, pSWS bicoherence = 0.017 ± 0.002 SD, pQAS bicoherence = 0.030 ± 0.015 SD, pREM/AS bicoherence = 0.018 ± 0.002 SD, p < 0.001, Bonferroni corrected). All analyses presented thus far were computed on global average rereferenced µECoG signals (see methods for preprocessing steps), a process that eliminates signal components common to all µECoG channels, such as movement artifacts or electrical noise. However, this preprocessing step may also eliminate physiological signals that are commonly measured across µECoG channels. To control for this, we recomputed bicoherence analyses for non-rereferenced µECoG signals. In addition to the previously mentioned bands involved in cross-frequency coupling, this analysis revealed bicoherence between theta and gamma frequencies that was strongest during the pREM/AS state (Supplementary Figure 3, pREM bicoherence = 0.042 ± 0.006 SEM, pSWS bicoherence = 0.024 ± 0.005 SEM, pQAS bicoherence = 0.030 ± 0.004 SEM). Therefore, each clustered brain state displays a characteristic pattern of cross-frequency interactions, delta/high gamma and delta/alpha coupling for the pSWS state, alpha/high gamma coupling for the pQAS state, and globally synchronous theta/gamma coupling for the pREM/AS state. Now that we have identified how signatures of spectral amplitude and local cross-frequency interaction fluctuate as animals transition between states, we next focus on investigating how patterns of large-scale cortical synchronization are reorganized across states.

### State dependent patterns of large-scale cortical synchronization

**Figure 6:**
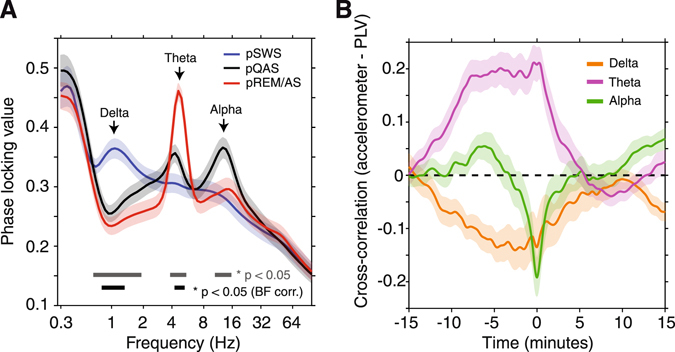
Spectral signatures of cortical synchronization across brain states. (**A**) Population average phase locking value (PLV) computed within each brain state (n = 23 sessions). PLV’s were computed between all pairs of µECoG channels to measure patterns of global cortical synchronization. PLV displayed significant state-dependent modulations in the delta, theta, and alpha frequency bands. Significant modulations are shown as bars at the bottom of the plot (gray bar – no correction for multiple comparisons, black bar – Bonferroni corrected p-values, n = 80 frequencies). (**B**) Population average cross-correation of accelerometer and PLV time series for the delta, theta, and alpha carrier frequencies (± SEM, n = 23). Note the strong posistive correlation of theta PLV up to 10 minutes prior to movement epochs. Conversley, alpha PLV is negatively correlated in a tight window around movement periods.

To identify the carrier frequencies of large-scale cortical synchronization, we computed the phase-locking value (PLV) between all pairs of µECoG channels for frequencies 0.3–100 Hz. Figure 6 displays the global average PLV spectrum across the three clustered brain states. The largest across-state differences were observed in delta, theta, and alpha frequency bands. Delta phase synchronization was strongest during the pSWS state (PLV = 0.37 ± 0.02 SEM), while pREM/AS and pQAS states were characterized by elevated theta (PLV = 0.46 ± 0.01 SEM) and alpha (PLV = 0.30 ± 0.02 SEM) synchronization, respectively (Fig. 6). Kruskal-Wallis analysis of variance confirmed that PLV in these frequency bands was significantly modulated across brain states (p < 0.05), however only changes in PLV for delta and theta frequency bands remained significant after Bonferroni correction (p < 0.05, n = 80 frequencies). Given that the strength and carrier frequency of large-scale synchronization is systematically modulated with changes in brain state, we next recomputed PLV in delta, theta, and alpha frequency bands in a time resolved manner using a one-minute sliding window. This window length was chosen to match the temporal structure of principal components, enabling an examination of fluctuations in PLV’s across brain state-space and with animal movement.

To assess how cortical synchronization co-varies with changes in animal movement, we computed the cross-correlation of accelerometer and PLV time series. This analysis revealed that movement epochs were preceded by prolonged periods of strong theta synchronization (corr = 0.21 ± 0.03 SEM), and weak delta synchronization (corr = −0.14 ± 0.05 SEM, Fig. 6, right). At the same time, animal movement was negatively correlated with alpha PLV time series in a relatively short window of ± 2 minutes around movement epochs (corr = −0.19 ± 0.04 SEM). Interestingly, accelerometer-PLV cross-correlation plots in all frequency bands converge on zero in the minutes following movement epochs, suggesting that movement has little predictive power over upcoming changes in cortical synchronization. In contrast, the relatively strong correlation of PLV with accelerometer time series up to 10 minutes prior to movement epochs suggest that measures of cortical synchronization may be predictive of upcoming movement.

**Figure 7:**
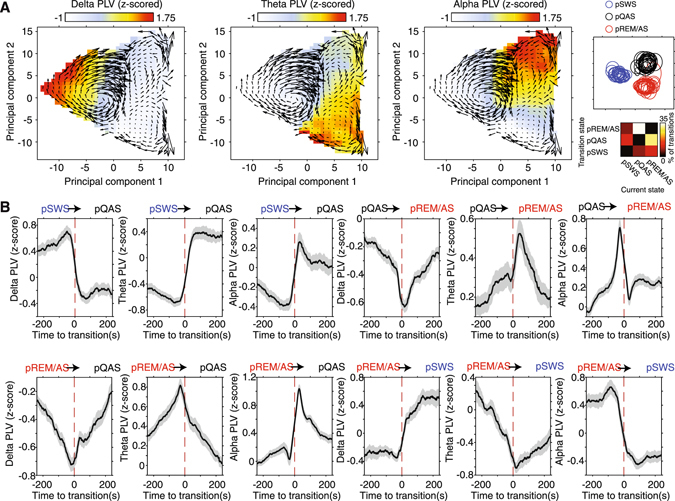
State-dependent fluctuations in cortical synchronization. (**A**) Population average PLV for delta (left), theta (middle), and alpha (right) frequency bands across the first two principal components of state space. In each plot population average state-space trajectories are overlaid to illustrate how animals transitioned between brain states. PLV’s were z-score normalized within session prior to computing mean PLV to account for differences in baseline connectivity between animals and sessions. The location of pSWS, pQAS, and pREM/AS clusters in state space is shown in the upper right for reference, while the frequency of state transitions is shown in the bottom left. Note that each clustered brain state is predominantly characterized by cortical synchronization in a different carrier frequency band. In addition, elevated PLV’s in the theta band extend from the pREM/AS cluster into the pQAS cluster. (**B**) Mean (± SEM) z-score normalized PLV’s time-locked to the most frequent state transitions. The identity of brain state transition is indicated above each plot, while PLV carrier frequency is labeled on the y-axis. Note that most state transitions are marked by distinct fluctuations in cortical synchronization across each carrier frequency band. In addition, transitions between pQAS and pREM/AS display more rapid reorganization of cortical synchronization than transitions to and from the pSWS state.

We next examined how PLV’s in the delta, theta, and alpha carrier frequencies fluctuated across PCA derived state space. Increases in the global mean of PLV’s in each carrier frequency were clearly localized to specific regions of state space, with delta PLV localizing to the pSWS state, theta PLV localizing in pREM/AS and extending into the pQAS, and alpha PLV predominantly localizing to the pQAS (Fig. 7A). To assess how PLV’s in identified frequency bands were modulated around specific brain state transitions, we computed time-resolved PLV’s in a window ± 240 seconds around each class of state transition. Since animals had varying baseline PLV’s for each recording session, PLV’s were z-score normalized within each recording prior to computing mean PLV’s time-locked to state transitions. The most commonly occurring state transitions were marked by a clear reorganization of global cortical synchronization across delta, theta, and alpha frequency bands (Fig. 7B). For example, the transition between pSWS and pQAS led to a sharp drop in delta PLV, paired with a sharp increase in theta and alpha PLV’s (Fig. 7B). The most frequent state transitions, from pREM/AS to pQAS and vice versa, revealed state-dependent fluctuations of PLV’s on a much shorter time-scale, particularly in the alpha band. These results reflect the rapid nature of transitions between pREM/AS and pQAS that are maximally separated along the second principal component state space axis.

To ensure that PLV results were not contaminated by measuring common sources across µECoG electrodes, we computed the imaginary part of coherence between all pairs of channels for each brain state. This crucial control analysis exclusively considers the non-zero phase lagged component of coherency, by definition mathematically excluding effects of volume conduction that occur with zero phase lag^21^. Imaginary coherence spectra were in agreement with PLV results, with clear peaks in the delta, theta, and alpha frequency bands (Supplementary Figure 4). These results indicate that changes in PLV’s observed across brain states are caused by variations in the phase consistency of physiological rhythms distributed across cortex, rather than measuring common sources across electrodes.

### Fragmentation and synchronization of cortical networks across brain states

**Figure 8:**
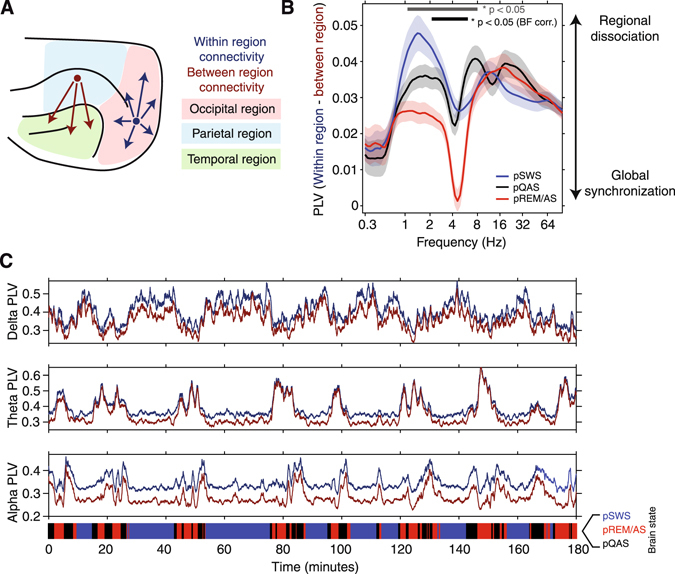
Comparison of short-range within-region, and long-range between-region functional connectivity. (**A**) µECoG electrodes were grouped into either occipital (red), parietal (blue), or temporal (green) regions based on the cortical area that was underlying each electrode. PLV was then computed for pairs of electrodes that fell within the same group (dark blue arrows) and pairs of electrodes that were between groups (red arrows). (**B**) Difference between within-region and between-region PLV, plotted as a function of carrier frequency. Large values of PLV_(within – between region)_ suggest that short-range connections dominate over long-range connections, indicating a dissociation of cortical regions. While small values of PLV_(within – between region)_ indicate that the strength of short-range and long-range connections is similar, inferring that the cortex is in a state of global synchronization. Note that long-range cortical synchronization is maximally fragmented during the pSWS state at the delta carrier frequency. In contrast, globally synchronized theta oscillations can be observed during the pREM/AS state. Significant state-dependent modulation of PLV_(within – between region)_ is shown as bars at the bottom of the plot (gray bar – no correction for multiple comparisons p < 0.05, black bar – Bonferroni corrected p < 0.05, n = 80 frequencies). (**C**) Strength of within-region PLV (dark blue) and between-region PLV (red), computed using a one-minute sliding window across an example recording from animal #2. The result of objective brain state classification is shown as a color bar at the bottom of the plot (pSWS – blue, pREM/AS – red, pQAS – black). State transitions lead to marked changes in patterns of local and long-range cortical synchronization. Note how within-region and between-region theta PLV traces converge and diverge as animals enter and exit the pREM/AS (middle plot).

Until now we have shown how patterns of global cortical synchronization are modulated across brain states. However, global mean PLV’s place an equal weight on both local and long range cortical connections. Brain networks are proposed to be organized according to a small-world topology, where dense local connections form network clusters, and a small number of long-range connections forming links between clusters^22^. Given these proposed organizational principles, we next set out to investigate how the brain balances the strength of local within-region connections, and long range between-region connections across brain states. For within-region connections we clustered µECoG recording electrodes that were positioned over occipital, parietal, and temporal cortical regions, respectively (Fig. 8A, blue arrows). Within-region functional connectivity was then defined as the mean PLV between all pairs of electrodes within each regional cluster. While between-region functional connectivity was defined as the mean PLV computed between all pairs of electrodes that did not belong to the same regional cluster (Fig. 8A, red arrows). To compare the relative strength of local and long range synchronization across brain states, we subtracted between-region PLV’s from within-region PLV’s. High values of PLV_(within − between region)_ indicate that local interactions are much stronger than long-range interactions, and by extension that brain dynamics are dominated by the existence of locally coherent but globally fragmented cortical networks. In contrast, low values of PLV_(within − between region)_ indicate that short-range and long-range synchronization are of similar strength, indicating that local dynamics are embedded within globally synchronized cortical networks. The population average PLV_(within − between region)_ spectrum across all four brain-states is shown in Fig. 8B. This analysis revealed that pSWS and pREM/AS states are characterized by two contrasting phenomena; the fragmentation of long-range cortical connections in the delta carrier frequency during pSWS, and the global synchronization of theta rhythms during pREM/AS. In contrast to the identified delta and theta bands, alpha band PLV_(within − between region)_ was relatively invariant across brain states (Fig. 8B, p > 0.05 Kruskal-Wallis test).

To illustrate how local- and long-range cortical synchronization evolves across multiple slep/wake cycles, Fig. 8C shows average within-region and between-region PLV time series in the delta, theta, and alpha frequency bands from an example recording. Reflecting the PLV_(within − between region)_ spectrum, the relative strength of measures of short- and long-range functional connectivity systematically fluctuate across brain states. For example, during the pSWS state within-region PLV in the theta band (blue trace, middle plot) was stronger than between-region PLV (red trace, middle plot). However, as animals transition to the pREM/AS state (via pQAS), both within- and between-region PLV traces converge such that the strength of short- and long-range measures of synchronization were almost equal, indicating a transition to a state of global theta synchronization (Fig. 8C, middle). Similarly, the fragmentation of cortical networks mediated by the delta carrier frequency can be observed as the divergence of within-module and between-module PLV traces during the pSWS state (Fig. 8C, top). Collectively, these results show that the relative strength of synchronization between local and distributed cortical networks dynamically reconfigures as the brain transitions between the various states of the resting/awake alternations.

## Discussion

Transitions in state are ubiquitous in the brain, however the consequences of such transitions for the organization of cortico-cortical functional connectivity remain unclear. To obtain an objective classification of brain state, we mapped time-resolved spectral properties into lower-dimensional state-space using principal component analysis. Brain-state clusters in state-space were consistent across all animals, with each animal following recurring trajectories through the states of the resting/awake alternations. Functional connectivity changed systematically across brain states, with largest changes occurring in the phase synchronization of delta, theta, and alpha oscillations. Presumed SWS state and REM/AS displayed contrasting modes of large-scale cortico-cortical functional connectivity, where pSWS was characterized by locally synchronized delta oscillations, and pREM/AS by globally synchronized theta oscillations. Collectively, our data provide strong evidence that large-scale functional interactions in the brain dynamically reconfigure across intrinsic brain states.

It has been repeatedly shown in the last 50 years that different states of vigilance in humans and animals are principally defined by specific patterns of EEG activity^1, 23, 24^. Brain states have classically been delineated using standard polysomnography, a method that combines information from electrocorticographic, electrooculographic, and electromyographic data modalities to classify sleep and wake states^25^. We employed a completely data-driven brain state classification method based solely on the time-varying nature of cortical LFP spectral properties. Similar data-driven approaches in mice have achieved a state classification accuracy of over 90%^26^. In contrast to classic brain state classification methods, which infer the existence of several discrete and independent states, our data support the view that major brain states exist as a continuum^18, 27^. Additionally, most of the major brain state clusters present in our data appeared to consist of a number of sub-states, which are best delineated by examining data trajectories in state-space. These observations suggest that the temporal evolution of brain state can be conceptualized as a multistable process, defined by a recurring set of neural conditions, with some stages displaying a strong degree of stability (e.g., pSWS), while others display a greater dynamical variability (e.g., pQAS and pREM/AS). Therefore, despite the convenience of treating brain states as independent and discrete, as most classical approaches entail, data reported here suggest that it may be more intuitive to quantify brain state by the temporal evolution of neuronal variables through state space.

Many previous studies on large-scale functional connectivity in the brain have computed connectivity measures across large temporal windows, with the implicit assumption that the dynamics of connectivity are stationary. While these studies have indeed led to breakthroughs such as the identification of resting state networks^2, 28, 29^ and rich club nodes^30, 31^ it is widely accepted that these approaches do not capture the true temporal dynamics of functional interaction in the brain^14, 15^. This is illustrated, for instance, by recent fMRI work showing that a third of human subjects fall asleep after 3 minutes in the scanner, with the global structure of hemodynamic signal correlations changing following the induction of sleep^11^. Apart from sleep, BOLD based functional connectivity has been shown to reorganize as a result of more subtle state transitions, such as engaging in cognitive tasks^32, 33^, learning^34, 35^, as well as more stark state transitions such as general anesthesia^36^. However, in contrast to such studies of BOLD functional connectivity, which mostly reveal correlated fluctuations in the amplitude of very slow neural dynamics^37^, we report here the state-dependent reorganization in phase synchronization of faster and more physiologically meaningful oscillations^38^.

Early studies on functional connectivity in the cat reported that delta oscillations were highly synchronous up to a distance of several millimeters in the cortex^39^. However, human ECoG studies during both SWS^40^, and anesthesia^7^, have shown that delta oscillations become increasingly asynchronous with greater inter-electrode distance. Although first appearing contradictory, the abovementioned studies strongly support our findings on short- and long-range functional connectivity during pSWS. Namely, we report that during pSWS delta oscillations are highly synchronous within cortical modules, but at the same time long-range inter-areal connections are weak, reflecting the functional fragmentation of widely distributed cortical networks. Such temporal and spatial disintegration of cortical functional connectivity during SWS and anesthesia is proposed to be the underlying cause for the loss of consciousness during these states^9, 41, 42^. In addition to state transitions between sleep and awake, the fragmentation of cortical networks has also been shown to manifest during pathophysiological transitions in brain state, such as the onset of epileptic seizures^43^. Indeed, abnormalities in the functional integration and fragmentation of cortical networks not only define the conditions for seizure onset, but also how cortical network dynamics propagate through a number of recurring seizure ‘sub-states’^44–46^.

In contrast to the fragmentated nature of delta cortical oscillations, theta oscillations tended to synchronize both short- and long-range connections in a state-dependent manner, with pREM/AS most strongly characterized by global theta synchronization. Despite displaying periods of widely synchronized activity within cortex, theta oscillations arise through reciprocal interactions between pacemakers in the medial septum, hippocampus, and entorhinal cortex^47^. One potential function of widespread cortical theta synchronization, as observed during pREM/AS, could be to temporally coordinate projections from the neocortex to the hippocampus, facilitating the replay and consolidation of recent memories during REM sleep^47–49^. Indeed, the globally synchronous theta/gamma cross-frequency coupling observed in cortical LFPs may represent the spectral fingerprint of such cortico-hippocampal information transfer^50, 51^.

The next steps should be towards a similar state-dependent examination of functional connectivity in humans with less spatially specific data modalities such as EEG and magnetoencephalography (MEG). Similar to results presented here for sleep/wake state transitions, it may be that the rapid reorganization of large-scale functional interaction represents the most informative neural signatures of the spontaneous drift of mental activity during wake resting periods. The continuing shift towards investigating and modulating large-scale neural dynamics within the context of intrinsically varying brain states will provide valuable insights into the neural mechanisms underlying consciousness, and will lead to the development of novel network-based tools for defining brain states under both normal and pathophysiological conditions.

## Materials and Methods

### Animals

Five adult female ferrets (*Mustela putorius furo*) were used for experiments (Euroferret, Dybbølsgade, Denmark). Each ferret was kept in a standard ferret cage Type 4541 P by Tecniplast (Hohenpeißberg, Germany) with an enriched environment under controlled ambient conditions (21°, 12-h light/dark cycle, lights on at 8:00 a.m.). The animals had ad libitum access to food pellets and to tap water. All behavioral testing was done during the light cycle between 12 a.m. and 6 p.m. All experiments were approved by the independent Hamburg state authority for animal welfare (BUG Hamburg) and were performed in accordance with the guidelines of the German Animal Protection Law.

### Custom µECoG design

We chose micromachining technology^52^ to design and develop a µECoG array that matches the anatomy of the ferret brain. The µECoG arrays had 64 platinum electrodes with a diameter of 250 µm, and were arranged in a hexagonal formation with an interelectrode distance of 1.5 mm. Thin-film metallization of electrodes and connection lines was sandwiched between two polyimide layers that served as substrate and insulation. Figure 1A displays a schematic diagram of the µECoG layout.

### ECoG implantation

Animals were initially anesthetized with an injection of a mix of ketamine (15 mg/kg), atropine (0.15 mg/kg) and medetomidine (0.08 mg/kg). Physiological parameters such as the ECG and rectal temperature were monitored throughout the surgery to maintain the state of the animal. To maintain anesthesia, additional ketamine (20 mg/kg) was given after each hour or on demand. All surgical procedures were performed under sterile conditions. After preparing the operating area, a craniotomy was performed over the left posterior cortex. The dura was carefully reflected, and the µECoG array was gently placed on the surface of the cortex such that it covered occipital, temporal and parietal areas (Fig. 1B). The dura was then folded back over the µECoG, and an absorbable artificial dura (Braun, Germany) was placed above the surgical area. The excised piece of bone was fixed back in place with titanium plates and screws, gaps were filled with fast set putty (Synthes, Germany). Finally, the µECoG interface was placed on top of the skull and fixed in place with dental cement. After the surgery the animals received preventive analgesics (carprofen, 4 mg/kg) and antibiotics (enrofloxacin, 5 mg/kg) for 8 days.

### Alignment of µECoG placement and parcellation of cortical areas

For each animal, the position of µECoG arrays over posterior cortex was recorded during surgery by taking photographs through a Zeiss OPMI pico microscope. The position of all 64 µECoG electrodes was then projected onto a scaled illustration of a model ferret brain. The precise position of a reference electrode on the scaled ferret brain was measured, along with the angle required to rotate the µECoG such that all electrodes aligned with the picture from surgery. A rotation matrix was then used to translate the location of all 64 µECoG electrodes onto the standard map of the ferret cortex^53^. The cortical region underlying each electrode was then noted, with regions clustered more generally into occipital, parietal, and temporal brain regions (Fig. 8A).

### Recording procedure

After recovery from surgery, ferrets were gradually accustomed to a recording box (45 × 20 × 52 cm) that was placed in a dark sound attenuated chamber. Once animals were ready for recordings, they were connected to a counter-balanced recording cable that was sufficiently long to enable them to move freely throughout the entire recording box. To monitor animal movement, an accelerometer was tightly attached to the cable-interface close to the head. Ferrets had no access to food or water during recording sessions. For each animal, we obtained at least 5 separate recording sessions that were longer than 2 hours. µECoG signals were digitized at 1.4 kHz (0.1 Hz high pass and 357 Hz low pass filters), and sampled simultaneously with a 64 channel AlphaLab SnR^TM^ recording system (Alpha Omega Engineering, Israel). From five animals, we analyzed a total of 23 recording sessions (recordings per animal: 5, 5, 4, 5, 4).

### Data Analysis

All data analysis was performed using custom scripts for Matlab (Mathworks Inc). To control for drifting and movement related artifacts, the global average signal across the entire µECoG grid was subtracted from each LFP time series. Noise epochs were detected using a threshold of 10 standard deviations. Data were rejected in a window of ± 10 seconds from all time points that exceeded this threshold. To compute time-resolved spectra, LFP signals were convolved with a series of 80 logarithmically spaced Morlet wavelets spanning frequencies 0.3–100 Hz. Spectral power was then computed by taking the square of the absolute value of time frequency estimates in the time domain. LFP spectrograms were then smoothed in the time domain using a Gaussian kernel of one-minute width (for comparison of smoothed and raw spectra PCA, see Supplementary Figure 1).

To project time-varying spectral properties into state space, we first z-scored the analytic amplitude of µECoG signals over time to normalize amplitude fluctuations across all frequencies (Fig. 2A, right). Principal component analysis was then performed on the z-scored amplitude matrix. The principal components that collectively explained more than 80 % of variance in spectra were used to construct the brain state space. Inspection of the principal component eigenvector coefficients as a function of LFP frequency revealed that principal components captured information spanning all frequency bands (Fig. 2C). To compute trajectories in state space we first took the first derivative of principal component time series to acquire the rate of change across each state space axis. Then the derivatives of the first two principal components were combined to generate state space velocity vectors for each data sample. Velocity vectors were binned according to location in state space, and the mean state space velocity vector for each bin was computed. Finally, mean velocity vectors were overlaid as topological ‘quiver’ plots on state space plots, where the direction and length of vectors indicate direction and rate of change through state space (for example, Fig. 3D).

To compute the optimal number of clusters across brain state space, we performed k-means cluster analysis assuming between 2 to 8 clusters (Fig. 3A). The optimal number of clusters was defined by computing the squared Euclidean distance between resultant data clusters. For each recording session, data from one electrode placed over visual cortical area 21 was used to project time-varying spectral properties into state space. To account for variance in clustering results based on differing initial cluster estimates, we repeated k-means clustering 100 times and took the most common cluster assignment for each data point.

### Bicoherence

Bicoherence is a normalized third order statistical moment to quantify cross-frequency coupling. In its most simple version, which is used here, it is a univariate measure estimating coupling within and not across sensors. It is defined as

1 $$b({f}_{1},{f}_{2})=\frac{\langle z({f}_{1})z({f}_{2}){z}^{\ast }({f}_{1}+{f}_{2})\rangle }{N}$$

In our implementation, *z*(*f*) is the Fourier transform of a Hanning windowed signal in a segment, and averaging is done across all segments. *N* is a normalization factor constructed from 3-norms of signals for each frequency as suggested in Shahbazi *et al.* (2014)^54^. In contrast to all other normalization, this normalization has the properties that a) the absolute value of bicoherence is bounded by one, and b) the normalization itself does not dependent on the cross-frequency coupling but only on the amplitude of signals for each frequency.

Note that bicoherence is a function of two frequencies even though it is a measure of coupling between three frequencies with the third frequency being the sum of the first two. The reason for constraining the third frequency is that for any other setting bicoherence vanishes for continuous or task related data recordings. For example, this is analogous to coherence, which is a function of one frequency even though it is a measure of a coupling between two signals. Linear coupling between signals of two different frequencies vanishes for continuous data.

### Functional connectivity analyses

To quantify phase synchronization between µECoG signals, we computed the phase locking value (PLV) across frequencies 0.3–100 Hz. Briefly, the instantaneous phase *θ* was extracted from analytic signals that were produced by convolving µECoG time series with complex Morlet wavelets. PLV between channels A and B at carrier frequency *f* was then defined by the following formula^55^

2 $$PL{V}_{AB}(f)=\frac{1}{N}|\sum _{n=1}^{N}{e}^{i({\theta }_{n}^{A}(f)-{\theta }_{n}^{B}(f))}|$$

PLV was computed between all pairs of µECoG electrodes. From subsequent PLV functional connectivity matrices we extracted three variables for each recording session: the global mean PLV, the average PLV of within-region channel pairs, and the average PLV of between-region channel pairs. State-dependent PLV was computed by pooling together all data samples from each clustered brain state for each recording session. In addition, we computed cortico-cortical PLV using a sliding window of one-minute duration with one second step size. This temporal scale of time-resolved PLV was chosen to match spectral smoothing prior to PCA, allowing a direct examination of how large-scale patterns of functional connectivity in the brain evolve through state space. Finally, to ensure that phase synchronization effects were not derived from measuring common sources across µECoG electrodes, we additionally computed the imaginary part of coherence^21^.

In addition to phase-based measures of functional connectivity, we also computed the amplitude envelope correlation of µECoG signals. Prior to computing amplitude correlation, time-frequency estimates from pairs of signals were orthogonalized to control for the effects of common sources being measured on both electrodes^2^. This crucial step removes zero phase–lagged components shared between simultaneously recorded µECoG signals, and ensures that only nonzero phase–lagged signal components are considered for amplitude envelope correlation analysis. Following orthogonalization, amplitude envelope time series were computed by taking the absolute value of analytic signals. The linear correlation coefficient was then computed between amplitude time series based on objective brain state classification.

### Statistics

All statistical tests were performed in MATLAB (Mathworks). To test if data conformed to a normal distribution, we performed Kolmogorov-Smirnov tests on each data distribution. Since most data violated the assumption of a normal distribution, non-parametric Kruskal-Wallis tests were used to quantify how neurophysiological and functional connectivity metrics differ between clustered brain states. Where applicable, Bonferroni correction was used to adjust p-values to account for multiple comparisons.

## Electronic supplementary material

Supplementary Information
