# Supplementary material for: Dynamic reconfiguration of cortical functional connectivity across brain states
Source: Sci Rep. 2017 Aug 18;7:8797. doi: 10.1038/s41598-017-08050-6 (PMC5562766; doi:10.1038/s41598-017-08050-6)
Supplement: Supplementary file 1 — Supplementary Information [file 41598_2017_8050_MOESM1_ESM.pdf]

Dynamic reconfiguration of cortical functional connectivity across brain states

Iain Stitt[*1,2], Karl J. Hollensteiner[*1], Edgar Galindo-Leon[1], Florian Pieper[1], Eva Fiedler[3], Thomas Stieglitz[3], Gerhard Engler[1], Guido Nolte[1], Andreas K. Engel[1]

[1] Department of Neurophysiology and Pathophysiology, University Medical Center Hamburg-Eppendorf, 20246 Hamburg, Germany

[2] Department of Psychiatry, Neuroscience Center, University of North Carolina, Chapel Hill, NC 27514, USA

[3] Department of Microsystems Engineering, University of Freiburg, 79110 Freiburg, Germany

[*] These authors contributed equally to this work

Correspondence should be addressed to: Dr. Iain Stitt

Department of Psychiatry, Neuroscience Center, University of North Carolina, Chapel Hill, NC 27514, USA. Email: iain@email.unc.edu

Abbreviated title*: State transitions and functional connectivity*

Number of supplementary figures: 4

Supplementary figures

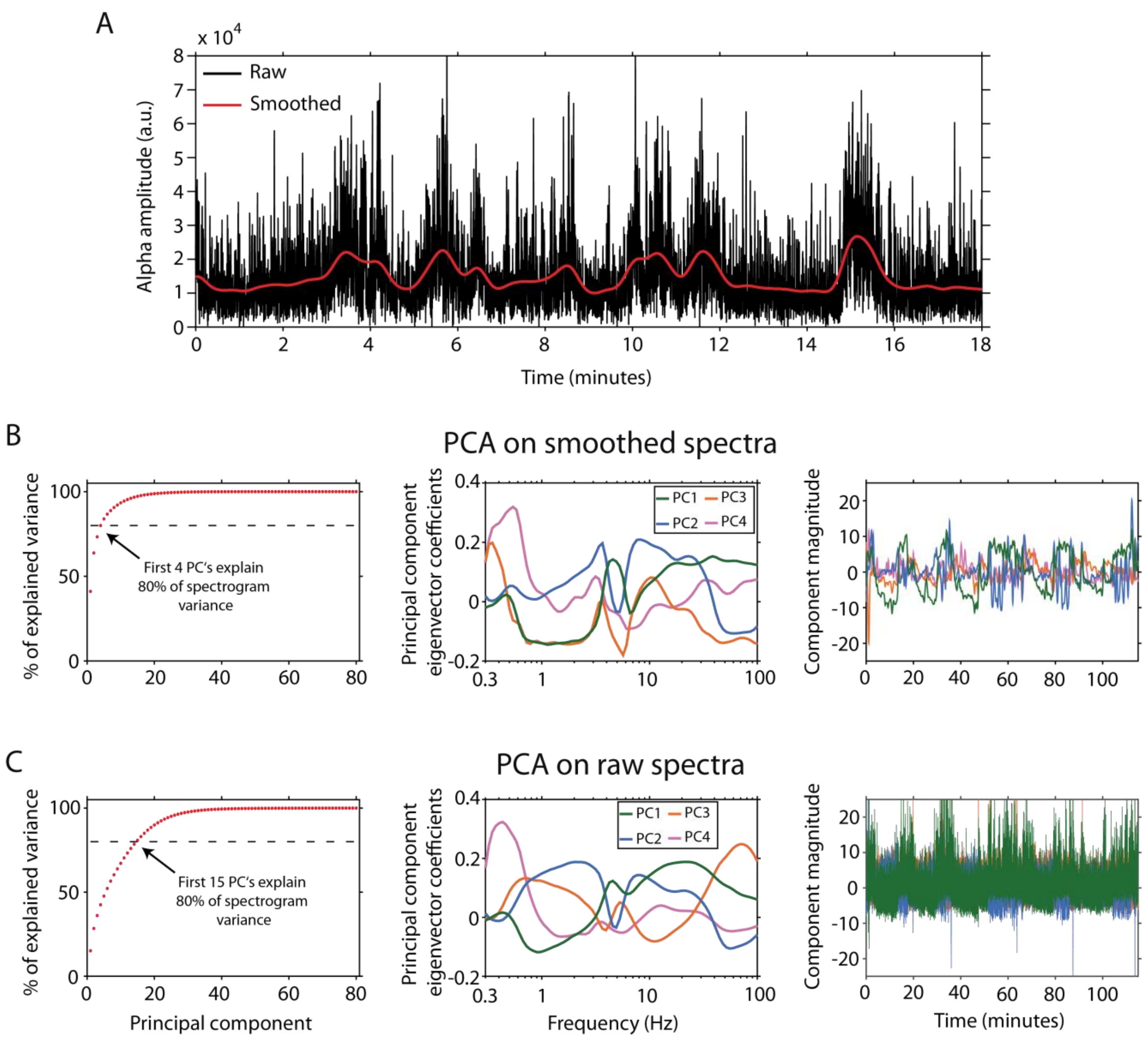

Supplementary Figure 1. Comparison of raw and smoothed spectra as inputs for principal component analysis. (A) Representative time series of raw alpha amplitude (black trace), and the same time series smoothed with a Gaussian kernel of 1-minute width (red trace). (B) Percentage of variance explained by each principal component, principal coefficient eigenvectors, and an example time series of the first four principal components for smoothed data. (C) Same as (B) but for data that were not smoothed prior to PCA. Note that far more components were required to reach the 80% variance threshold, and that raw traces of each component appear noisier.

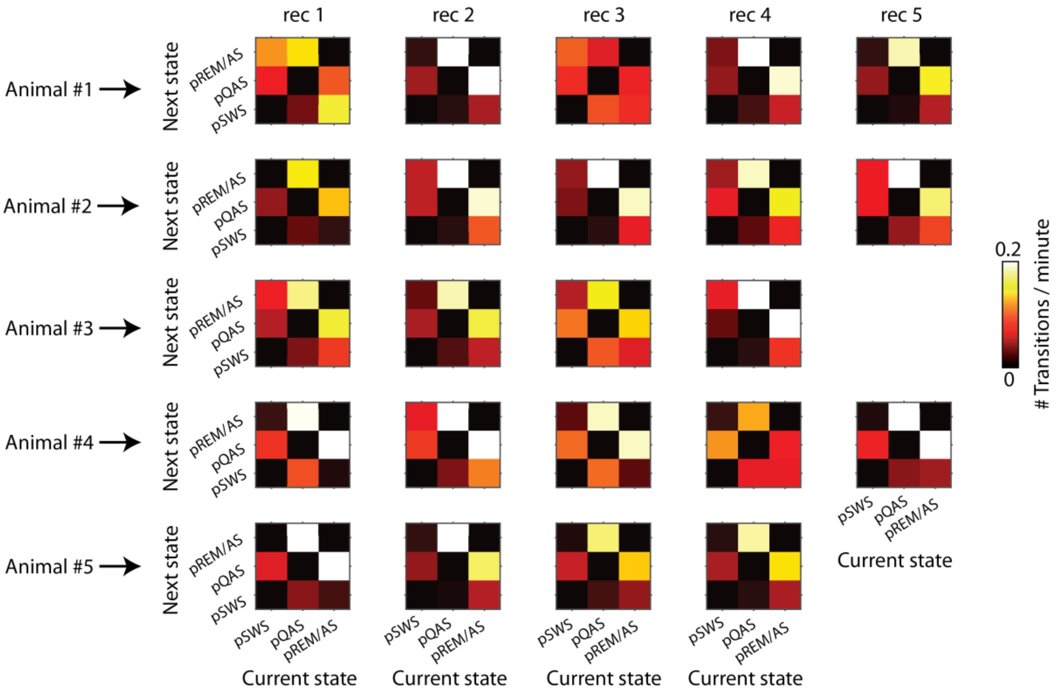

Supplementary Figure 2. Frequency of state tansitions across all sessions and animals. Each plot shows the frequency of transitions between each clustered state across individual recording sessions. Sessions from each animal are organized into rows.

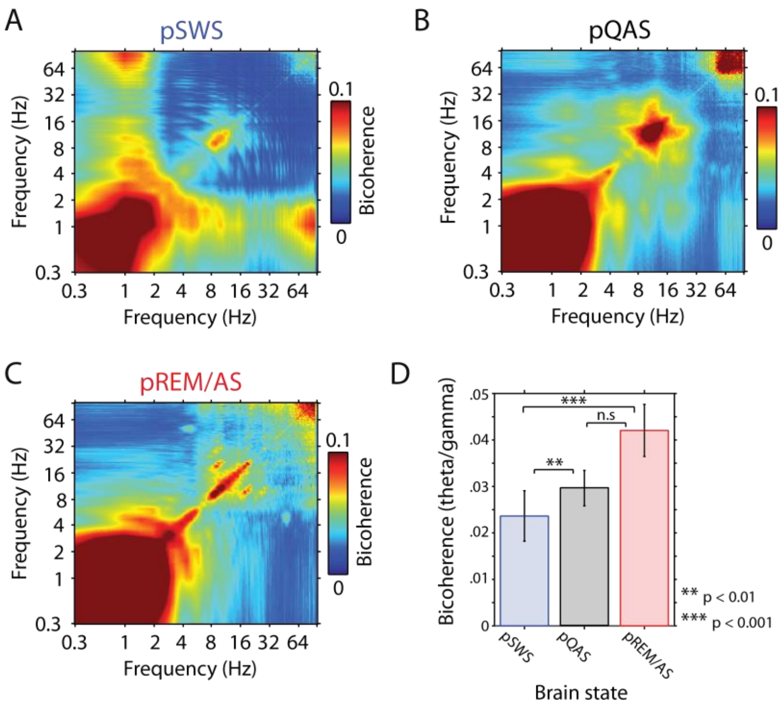

Supplementary Figure 3. State dependent changes in bicoherence without global average rereferencing. (A-C) Display bicoherence spectra computed on µECoG signals that were not rereferenced by the global mean µECoG signal. Note the presence of previously identified frequency-frequency bands, such as delta / high gamma coupling. Bicoherence in the pREM/AS (C) displays a clear peak in the theta / gamma range (4-5 Hz / 40-60 Hz) that was not present in globally rereferenced signals. (D) Bar graphs (± SEM) show the strength of theta / gamma bicoherence across each brain state (** $p < 0.01$, *** $p < 0.001$, kruskal-wallis test, Bonferroni corrected p-values).

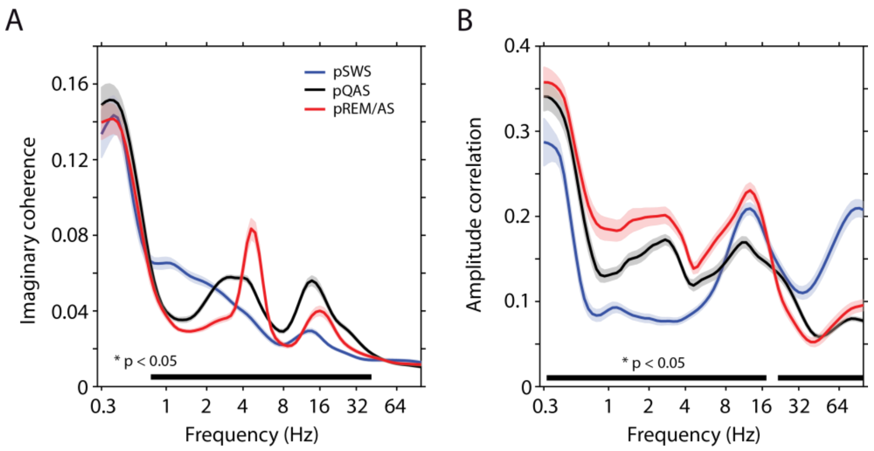

Supplementary Figure 4. Imaginary coherence and amplitude envelope correlation across brain states. (A) Population average imaginary coherence spectra across the three brain states. Note the significant state-dependent changes in imaginary coherence in the delta, theta, and alpha frequency bands, reflecting PLV results. These results show that state-dependent shifts in cortical synchronization, as measured by PLV, occur with non-zero phase lag, and are therefore not the result of measuring common sources across μECoG electrodes. (B) Population average amplitude envelope correlation spectra across brain states. Prior to computing amplitude correlation, time-frequency estimates of pairs of channels were orthogonalized to eliminate the contribution of common sources measured at each electrode (see methods). Peaks in amplitude envelope correlation spectra can be observed at the delta, alpha, and high gamma frequencies. Interestingly, despite displaying strong phase synchronization in the theta frequency band, this band lacked a clear peak in amplitude correlation spectra across brain states.
